# Dilution-Induced Deposition of Concentrated Binary Mixtures of Cationic Polysaccharides and Surfactants

**DOI:** 10.3390/polym15143011

**Published:** 2023-07-12

**Authors:** Laura Fernández-Peña, Eduardo Guzmán, Teresa Oñate-Martínez, Coral Fernández-Pérez, Francisco Ortega, Ramón G. Rubio, Gustavo S. Luengo

**Affiliations:** 1 Departamento de Química Física, Facultad de Ciencias Químicas, Universidad Complutense de Madrid, Ciudad Universitaria s/n, 28040 Madrid, Spain; laura.fernandez.pena@ucm.es (L.F.-P.); teresaon@ucm.es (T.O.-M.); cferna01@ucm.es (C.F.-P.); fortega@quim.ucm.es (F.O.); 2 Centro de Espectroscopía y Correlación, Universidad Complutense de Madrid, Ciudad Universitaria s/n, 28040 Madrid, Spain; 3 Instituto Pluridisciplinar, Universidad Complutense de Madrid, Paseo Juan XXIII 1, 28040 Madrid, Spain; 4 L’Oréal Research and Innovation, 1 Avenue Eugène Schueller, 93600 Aulnay-Sous-Bois, France

**Keywords:** conditioning, cosmetic, deposition, dilution, hair, precipitation, polyelectrolyte, shampoos, solid surface, surfactant

## Abstract

This work investigates the effect of dilution on the phase separation process of binary charged polysaccharide–surfactant mixtures formed by two cationic polysaccharides and up to four surfactants of different nature (anionic, zwitterionic, and neutral), as well as the potential impact of dilution-induced phase separation on the formation of conditioning deposits on charged surfaces, mimicking the negative charge and wettability of damaged hair fibers. The results obtained showed that the dilution behavior of model washing formulations (concentrated polysaccharide–surfactant mixtures) cannot be described in terms of a classical complex precipitation framework, as phase separation phenomena occur even when the aggregates are far from the equilibrium phase separation composition. Therefore, dilution-enhanced deposition cannot be predicted in terms of the worsening of colloidal stability due to the charge neutralization phenomena, as common phase separation and, hence, enhanced deposition occurs even for highly charged complexes.

## 1. Introduction

Cosmetic cleansing formulations, including gels and shampoos, consist of very complex multi-component mixtures containing, among many other components, at least one cationic polymer and several surfactants of different types (anionic, zwitterionic and neutral, and, in some cases, cationic as substitutes for cationic polymers) [1]. The combination of cationic polymers and surfactants gives a dual role to washing formulations. In fact, surfactants are cleansing agents that help remove dirt, oil, and residues from the hair, while cationic polymers, on the other hand, are used to condition and protect the hair [2,3]. Furthermore, when used together, surfactants and polyelectrolytes lead to the formation of supramolecular aggregates via various types of interactions (commonly electrostatic, hydrophobic, or hydrogen bonding) [4,5,6]. These aggregates are of paramount importance in the efficacy of hair care products to achieve clean, healthy, and manageable hair [7].

From a physicochemical point of view, cosmetic cleansing formulations are one-phase systems (1ϕ) that undergo a phase-separation process during the rinsing, resulting in the formation of stable coacervates, i.e., two-phase mixtures (2ϕ), which are deposited on hair fibers upon dilution during practical use [8,9,10]. Thus, the deposition of colloidal aggregates from the aqueous phase onto the keratinized surface of the hair fibers plays a central role in the final performance of shampoo formulations [11,12,13,14]. Therefore, the mechanism of action of shampoos is based on enhanced deposition driven by dilution-induced precipitation, the Lochhead effect, which results in the deposition of conditioning cationic polymers when the shampoo is rinsed off [15]. Understanding the mechanisms that govern dilution-induced phase separation and how to control it is a major challenge in achieving the ultimate efficacy of washing formulations [16]. This is essential to meet one of the key challenges facing the cosmetics industry in its transition to more environmentally friendly products. This requires the replacement of certain ingredients that do not meet current requirements for biodegradability and reduced environmental impact. In particular, the cosmetics industry is seeking alternatives to replace conventional cationic polyelectrolytes, commonly used as conditioning agents, with other compounds that meet the above requirements, including biopolymers or bio-based polymers [17]. However, to date, there have been few studies investigating model-cleansing formulations under practical conditions, e.g., under conditions that mimic the dilution process under the shower and the vectorization of deposits. This makes it very difficult to predict the performance of specific mixtures, which is essential for optimizing formulations containing new environmentally friendly ingredients.

In a previous study [18], the performance of concentrated polyelectrolyte–surfactant mixtures with a wide range of compositions was investigated in terms of the effect of dilution on their phase behavior and adsorption on negatively charged solid surfaces [2,19]. This present work aims to extend the previous study by analyzing the behavior of concentrated binary polyelectrolyte–surfactant mixtures in which the cationic polyelectrolytes are of natural origin or at least derived from natural products (bio based). This is a very important issue because the substitution of ingredients must lead to new formulations with similar or even better performance than those containing raw materials derived from petrochemical processes while at the same time increasing the biodegradability and reducing the eco-toxicity of the final products. Therefore, understanding the physicochemical basis of the performance of the model binary polyelectrolyte–surfactant mixtures can play a very important role in optimizing the formulation for specific applications using an appropriate combination of environmentally friendly ingredients [20].

## 2. Materials and Methods

### 2.1. Chemicals

Poly(diallyl-dimethylammonium chloride), PDADMAC, with a molecular weight in the range 100–200 kDa was purchased from Sigma-Aldrich (Saint Louis, MO, USA). Hydroxyethylcellulose quaternized with 2,3-epoxypropyltrimethylammonium chloride, JR400, and hydroxypropyl guar hydroxypropyltrimonium chloride, Jaguar C162, both with molecular weights in the range 500–600 kDa, was supplied by Dow Chemical Company (Midland, MI, USA) and Solvay Novecare (Princeton, NJ, USA), respectively. The polymers were used as received without further purification.

Four different surfactants were tested in the preparation of single-phase binary concentrated polyelectrolyte–surfactant mixtures, which can be considered as a model cosmetic washing formulation. Laureth-5 carboxylic acid (AKYPO) and sodium lauryl ether sulfate (SLES) were supplied by Kao Chemicals Europe (Barcelona, Spain). Cocamidopropyl betaine (CAPB) was purchased from Solvay Novecare (Princeton, NJ, USA). A mixture of caprylyl glucoside and capryl glucoside (APG) with equal molar fractions of both components was purchased from Safic-Alcan (Barcelona, Spain). Detailed information on the molecular formula of the cationic polyelectrolytes and the surfactants used in this work can be found in our previous publications.

The ultrapure deionized water used for cleaning and solution preparation was obtained from aquaMAX^TM^-Ultra 370 Series multi-cartridge purification system (Young Lin Instrument, Co., Anyang, South Korea). The water used had a resistivity higher than 18 MΩ∙cm and a total organic content lower than 6 ppm. Citric acid (purity 99.9%) and NaCl (purity 99.95%) purchased from Sigma-Aldrich (Saint Louis, MO, USA) were used to adjust the pH and ionic strength of the polyelectrolyte–surfactant mixtures.

### 2.2. Preparation of the Samples

Taking as a reference the composition of the mixtures studied in [18], different concentrated binary polyelectrolyte–surfactant mixtures were investigated. Table 1 shows the concentration of the different components in the binary concentrated polyelectrolyte–surfactant mixtures.

The polyelectrolyte–surfactant mixtures were prepared according to a protocol adapted from our previous publications [21]. First, the amount of polymer required to obtain final mixtures with a concentration of 0.5% *w*/*w* was weighed and poured into a flask. Then, the amount of solid NaCl (purity 99.95%) required to obtain a final concentration of 120 mM in the target mixtures was weighed and added to the flask. The final step was the addition of the surfactant from a stock solution (concentration twice that of the surfactant in the final polyelectrolyte–surfactant mixture) at pH~4.5, followed by dilution of the mixture with a citric acid solution at pH~4.5 to obtain the desired mixture composition. To prepare the mixtures, the components were added sequentially, and the solutions were homogenized overnight under mild stirring conditions (1000 rpm) [22,23,24,25]. All samples were aged at 25 °C for one week prior to use. Since citric acid is not a buffer, the pH was measured after the aging period and just before the samples were used to ensure that the samples were always measured at pH~4.5. All experiments were carried out at 25.0 ± 0.1 °C.

It is worth noting that the values of pH and ionic strength used in this study are in the range of those used in most hair care cosmetic treatments to reduce the bleaching of hair fibers [2,26]. Therefore, it is expected that the results obtained here will contribute to the understanding of the phenomena that occur during the dilution of shampoos during shower application.

### 2.3. Method

Turbidity of polyelectrolyte–surfactant mixtures was determined from transmittance (T) measurements at 400 nm using a UV/visible spectrophotometer (HPUV 8452). Turbidity was expressed as turbidity = 100 − T (%).

A quartz crystal microbalance with dissipation monitoring (QCM-D) from KSV (model QCM Z-500, Espoo, Finland) equipped with gold-coated AT-cut quartz crystals (note: gold-coated AT-cut quartz crystals were first cleaned with piranha solution, 70% sulphuric acid/30% hydrogen peroxide, for thirty minutes and then thoroughly rinsed with Milli-Q water) was used to evaluate the adsorption of polyelectrolyte–surfactant layers. The surface of the quartz sensor was modified using a self-assembled monolayer of 3-mercapto-propanesulfonic acid to obtain a negatively charged surface. QCM-D measures the impedance spectra of a quartz crystal for the fundamental frequency (f_0_ = 5 MHz) and the odd overtones up to the 11th (central frequency of the 11th overtone, f_11_ = 55 MHz). The impedance spectra obtained were analyzed using a single-layer model according to the procedure described by Voinova et al. [16], which provides the effective acoustic or hydrodynamic thickness of the adsorbed layer, *h*_ac_. This procedure allows the changes in resonant frequency Δ*f* and dissipation factor Δ*D* of the different overtones to be correlated with the physical parameters of the layers (thickness *h*_j_, density *ρ*_j_, elasticity *μ*_j_, and viscosity *η*_j_). Further details of the data analysis can be found in our previous publication [27].

An imaging null-ellipsometer from Nanofilm (model EP3, Göttingen, Germany) was also used to determine the amount of material adsorbed on the solid surfaces as optical thickness, *h*_op_. Ellipsometry experiments were carried out using a solid–liquid cell at a fixed angle of 60° with silica plates as substrates (Siltronix, Archamps, France). These substrates were treated with piranha solution for 30 min to create a charged surface similar to that of thiol-decorated gold surfaces [28]. The experimental variables measured via ellipsometry are the ellipsometric angles, Δ and *Ψ*, which are related to the ratio between the reflection coefficients for the parallel (*r*_p_) and normal (*r*_s_) components of the magnetic field derived by Fresnel, i.e., to the ellipticity *ρ*^e^. The optical thickness, *h*_op_, and refractive index, *n*, of the adsorbed layers are obtained from the experimental measurements by assuming a slab model describing the system. In this study, a four-layer slab was utilized. The first layer represents the silicon substrate with a refractive index of *n* = 4.1653 − 0.049i. The second layer corresponds to the native oxide layer, with a refractive index of *n* = 1.4653. The thickness of the oxide layer was determined by measuring the bare silicon wafer in water. The outermost layer (fourth layer) of the model was the solution with a constant refractive index similar to that of the polymer solution (*n* = 1.33). The third layer corresponds to the adsorption layer. The thickness and refractive index of the adsorption layers were determined by minimizing the differences between the experimental values of the ellipsometric angles and the results obtained from solving Fresnel’s equation using the four-layer model [27,29].

*h*_ac_ and *h*_op_ should not be considered absolute thicknesses due to the heterogeneity of most of the polyelectrolyte layers. Therefore, all discussions in this paper consider *h*_ac_ and *h*_op_ as effective thicknesses that provide different information about the amount adsorbed within the layer [29,30]. The combination of ellipsometry and QCM-D is important because of the different sensitivities of these techniques to the water. This is because while QCM-D provides information on the total mass of the adsorbed layer, including both the polymer and the water associated with such layer, ellipsometry, which is based on the differences between the refractive indices of the layer and the medium, only provides information on the amount of adsorbed polymer (or polymer–surfactant complexes). This difference leads to *h*_op_ ≤ *h*_ac_ and allows the water content of the layers *x*_w_ to be estimated as follows [21,28]:

(1) $$x_{w}=\frac{h_{ac}-h_{op}}{h_{ac}}.$$

AFM measurements of dry layers deposited on SiO_2_ substrates were carried out in air at room temperature using an NT-MDT Ntegra Spectra (NT-MDT, Russia) in the tapping mode with a silicon tip, model RTESP (Veeco Instrument Inc., Plainview, NY, USA). It is worth noting that even though some changes in morphology might be expected due to the drying process, the general aspects obtained from the analysis of wet and dry samples should not significantly alter the conclusions drawn from the images [27].

The model surfaces used in this present study are negatively charged and have a ξ-potential value of around −42 ± 5 mV (obtained from the measurement of the ξ-potential of colloidal particles with the same surface nature as the flat model surfaces [2]). This value is in good agreement with those found for the ξ-potential of damaged hair fibers, which ranges from −55 to −35 mV, depending on their origin and chemical treatment [2]. Therefore, model surfaces can be used to mimic the hair fiber surface, at least from a physicochemical point of view.

## 3. Results

### 3.1. Phase Separation on Dilution for Concentrated Binary Polymer–Surfactant Mixtures

The first part of this work attempts to clarify whether the concentrated binary polyelectrolyte–surfactant mixtures with the compositions given in Table 1 are stable, i.e., whether they are single-phase mixtures, and which is the behavior of the concentrated single-phase mixtures on dilution. This requires the study of twelve independent polyelectrolyte–surfactant pairs, corresponding to four polymer–surfactant pairs for each polymer considered. Table 2 shows the single-phase or phase-separated (2ϕ) character of the different concentrated binary polyelectrolyte–surfactant mixtures studied in this work.

The mixture of the three cationic polyelectrolytes with the four surfactants does not always lead to the formation of single-phase systems (1ϕ), and some of the mixtures undergo rapid phase separation immediately after mixing, i.e., they lead to the formation of 2ϕ systems. This is the case for the mixtures of the three cationic polyelectrolytes with the anionic surfactants (AKYPO and SLES). On the other hand, regardless of the polymer considered, the mixtures with the zwitterionic surfactant (CAPB) or the neutral one (APG) lead to the formation of 1ϕ systems. It should be noted that the cationic polyelectrolytes and the surfactant are completely soluble in water under the conditions used to prepare the binary mixtures.

The occurrence of phase separation in the mixtures containing the anionic surfactant cannot be understood by considering an equilibrium framework for the polyelectrolyte–surfactant association [11]. Thus, considering an electrostatic binding of the SLES or AKYPO to the positively charged residues of the polyelectrolyte and assuming that the surfactant concentration is several times higher than that of the charged monomer (in the case of mixtures containing PDADMAC, the surfactant/monomer ratio is about 6 and 4 for mixtures with SLES and AKYPO, respectively; this ratio increases up to 22 and 16 in the case of the mixtures of the cationic polysaccharides with SLES and AKYPO, respectively), the formation of soluble overcharged negative complexes should be ensured via the association of surfactant micelles with the polymer backbone. Therefore, the mixtures should have a single-phase character. However, this is not compatible with the results obtained, which could be related to the non-equilibrium nature of the association process [11]. In fact, the existence of compositional gradients during the preparation of the mixtures leads to Marangoni stresses that drive the formation of kinetically trapped aggregates with a lack of stability, even though their composition corresponds to a single-phase region [8,31].

**Figure 1:**
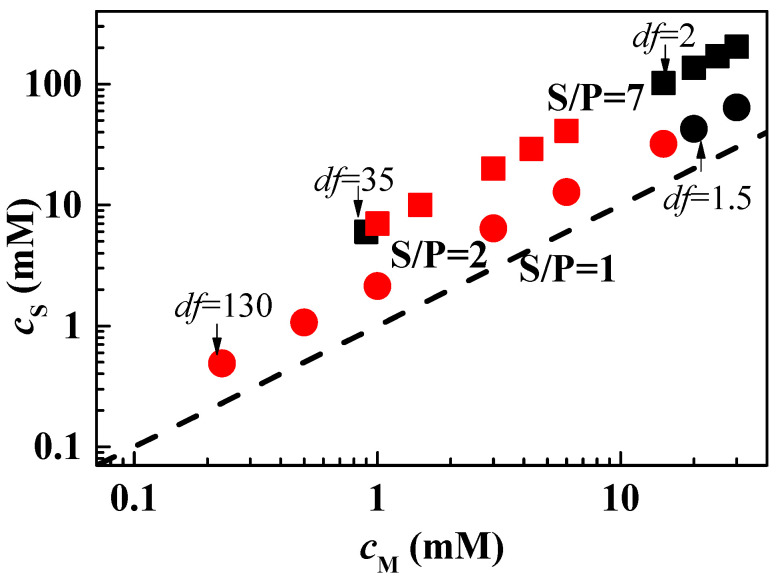
Surfactant (*c*_S_)-monomer (*c*_M_) concentration phase diagrams for the combination of PDADMAC with CAPB (■ and ■) and APG (● and ●). The black and red symbols correspond to one-phase and phase-separate mixtures, respectively and the discontinuous solid line corresponds to the dilution of a hypothetical mixture with S/P = 1. The values of the dilution factors (*df*) indicated in the plots correspond to the onset and exit on the phase separation region or the maximum *df* explored. Furthermore, the S/P values corresponding to the dilution line of each sample are also included. Data of PDADMAC-CAPB mixtures were adapted from the work by Fernández-Peña et al. [18] under CC-BY license.

Once the character of the concentrated mixtures has been defined. The next step is to evaluate the dilution behavior of the 1ϕ mixtures to determine the composition at which they reach the onset of phase separation. For this purpose, it is convenient to define the dilution factor (*df*), which indicates the number of times the original sample has been diluted with the addition of an aqueous NaCl solution (120 mM) at pH~4.5. In the case of the mixture of PDADMAC with CAPB and APG, very different behavior is found depending on the nature of the surfactant, which is not surprising given the different nature of the interactions involved in the PDADMAC–surfactant association. The association between PDADMAC and CAPB is expected to occur via electrostatic interactions between the terminal anionic carboxylic group in the polar head of the surfactant and the positively charged monomers of PDADMAC. Despite the electrostatic origin of this association process, the net charge of the complexes formed will remain similar to that of the naked polymer because each surfactant molecule that electrostatically binds to PDADMAC brings a positive charge (remember that CAPB is a zwitterionic character); therefore, the association does not result in effective charge neutralization [32]. However, at concentrations as high as those studied here, the association is not expected to occur via the binding of single surfactant molecules, but rather the complexes will be formed via CAPB micelles attached to the PDADMAC chain, leading to the formation of overcharged negatively charged complexes according to the results by Akanno et al. [32]. On the other hand, the association between PDADMAC and APG occurs via van der Waals interactions, involving the hydrophobic tail of the surfactant molecules and the hydrophobic domains in the polymer backbone. At the high concentrations studied here, this leads to the formation of complexes where APG hemimicelles are associated with the PDADMAC chain [33]. Figure 1 shows the phase diagrams of the dilution process for the mixtures of PDADMAC with APG, as a plot of the surfactant concentration, *c*_S_, versus the monomer unit concentration, *c*_M_. For the sake of completeness in the discussion, the data for PDADMAC-CAPB mixtures obtained in our previous publication [18] are also included. For comparison, Figure 1 shows the line corresponding to the dilution of a hypothetical mixture with S/P = 1 (S/P defines the ratio between the number of surfactant molecules and polymer monomers) and, where possible, the values of the dilution factor (*df*) corresponding to the limits of the phase separation region.

The results for the mixtures of the PDADMAC with CAPB and APG show some important differences. Although, in both cases, dilution factor (*df*) values around 2 drive the system to phase separation, the behavior of the mixtures with increasing dilution factor is different. In fact, in the case of mixtures with CAPB, the diluted mixtures recover their single-phase character at a dilution factor of around 35, whereas PDADMAC-APG mixtures remain phase separated even at a dilution factor of around 130. In light of the results obtained, for concentrated polyelectrolyte–surfactant mixtures, phase separation can occur on dilution guided for different physicochemical forces that the typical phase separation occurring via the neutralization of the polyelectrolyte charges as a result of the binding of an opposite surfactant. Indeed, in the dilution line for PDADMAC-CAPB and PDADMAC-APG mixtures, the S/P ratios remain at values seven and two times higher, respectively, than the neutrality line (S/P = 1). On the other hand, for PDADMAC-APG mixtures, no process can be expected to drive the system towards electroneutrality on dilution. Therefore, elucidating the origin of dilution-induced phase separation in polyelectrolyte–surfactant mixtures remains a challenge [34,35,36,37,38].

Focusing now on the mixture of PDADMAC with CAPB, the phase separation in dilution can be explained as a result of a combination of the reduced effective charge of the complexes, associated with the charge-screening phenomenon produced by the high ionic strength used (the dilution is carried out maintaining the ionic strength) and the changes in the degree of dissociation of the carboxyl groups during the dilution process. This may shift the charge neutralization line to higher surfactant concentrations, i.e., phase separation may start at S/P values far from unity [14]. In addition, considering that dilution takes place under conditions of ionic strength maintenance, an increase in the number of complexes and an increase in the attractive interactions between such complexes can be expected. This favors the association between individual complexes, regardless of their charge, and thus leads the systems to phase separation, in agreement with the results of Xu et al. [37]. This latter framework also justifies the appearance of phase separation on dilution in the PDADMAC-APG system.

**Figure 2:**
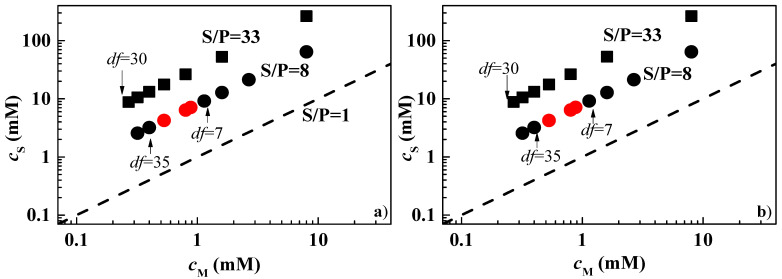
Surfactant (*c*_S_)-monomer (*c*_M_) concentration phase diagrams for the combination of JR400 (**a**) and Jaguar C162 (**b**) with CAPB (■) and APG (● and ●). The black and red symbols correspond to one-phase and phase-separate mixtures, respectively and the discontinuous solid line corresponds to the dilution of a hypothetical mixture with S/P = 1. The values of the dilution factors (*df*) indicated in the plots correspond to the onset and exit on the phase separation region or the maximum *df* explored. Furthermore, the S/P values corresponding to the dilution line of each sample are also included.

Understanding how dilution-induced phase separation proceeds in mixtures containing PDADMAC is of interest due to its extensive use as a conditioning polymer in a wide range of conditioning formulations. However, the aim of this work is to gain some physical insight into the behavior of cationic polysaccharides, which may be an alternative to PDADMAC. To this aim, dilution studies were carried out on concentrated mixtures of JR400 and Jaguar C162 with various surfactants. However, again, only mixtures with CAPB and APG were stable, and their behavior on dilution is analyzed below. Figure 2 shows the phase diagrams of the dilution process for the mixtures of JR400 and Jaguar C162 with the two surfactants considered, as a plot of the surfactant concentration, *c*_S_, versus the monomer unit concentration, *c*_M_.

The interpretation of the results for mixtures of JR400 and Jaguar C162 with the two surfactants considered is less straightforward than the results obtained for mixtures containing PDADMAC. In the case of mixtures with JR400 and Jaguar C162, in addition to the polyelectrolyte–surfactant interactions observed for the above mixtures with PDADMAC, the role of hydrogen bonding must be considered. Indeed, the polysaccharide nature of JR400 and Jaguar C162 allows the formation of many hydrogen bonds with surfactant molecules and solvents. Therefore, in the case of the mixtures of the cationic polysaccharides with CAPB, association in the concentrated state can be expected to occur via a combination of hydrogen bonding between the non-dissociated carboxyl groups forming CAPB micelles and the hydroxyl groups in the polymer backbone and electrostatic interactions between dissociated carboxyl groups and the ammonium groups of the polysaccharides. This results in hydrophilic complexes that remain solubilized in the aqueous medium. The dilution of the concentrated mixtures reduces the contribution of hydrogen bonding from the CAPB molecules to the cationic polysaccharides but increases the number of hydrogen bonds between the polysaccharide and the solvent, which ensures the stability of the aggregates regardless of the dilution factor. It is true that the contribution of the tail–tail interactions cannot be neglected, as was the case for the mixtures of PDADMAC and CAPB. However, this contribution seems to be sufficiently counterbalanced by the many hydrogen bonds that can be formed between the solvent and the polysaccharides to avoid phase separation, at least for dilution factors up to 30.

The situation is slightly different for the mixtures of the polysaccharides with APG, where only the hydrogen bonds contribute to the polymer–surfactant association. Therefore, it can be expected that the dilution process may favor the hydrophobic interactions between the tails of the surfactant molecules attached to different points of the polysaccharide chains; therefore, the dilution may contribute to bringing the systems towards a phase separation region, as occurred in the mixtures with PDADMAC [37].

**Figure 3:**
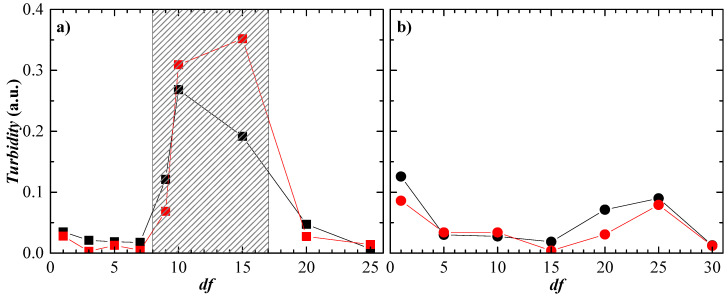
Dependence of turbidity on the dilution factor for the different polysaccharide–surfactant binary mixtures studied. (**a**) Polysaccharide–APG mixtures (■ Jaguar C162 ■ JR400). (**b**) Polysaccharide–CAPB mixtures (● Jaguar C162 ● JR400).

Figure 3 shows the dependence of turbidity on the dilution factor for mixtures of the two polysaccharides with CAPB and APG. The results confirm that the component defining the behavior of these mixtures is the surfactant, i.e., if the polysaccharide–surfactant mixtures have the same surfactant, they behave similarly regardless of the type of polysaccharide used. It should be noted that low turbidity values indicate that the samples are either in a single-phase equilibrium state or in a region of equilibrium between two phases where aggregates have already precipitated to form a sediment. A significant increase in turbidity, on the other hand, indicates that the samples are approaching the phase separation region due to the formation of insoluble aggregates or are directly entering a coacervation regime rather than a precipitation regime, as is the case here. Once the coacervation regime is overcome, the systems leave the phase separation regime and return to a single-phase regime characterized by its low turbidity. It should be noted that the aging of the mixtures containing APG leads to the precipitation of the complexes formed in the dilution factor range corresponding to coacervation (dilution factor values in the range 8–18). However, for the mixtures with CAPB, neither the turbidity results shown in Figure 3 nor the aging of the mixtures over a period of two months show any signature for the appearance of the phase separation region in the dilution factor range studied. It should be noted that the small increases in turbidity observed (see Figure 3b) are within the expected limits of error for turbidity determination; therefore, it is not physically meaningful to base any discussion on them. It is important to note that the results obtained for polysaccharide–CAPB mixtures do not imply that such mixtures do not have a phase separation region but that if it does occur, it may occur at higher dilution factors or at higher concentration conditions.

### 3.2. Study of the Deposition of Concentrated Binary Polyelectrolyte–Surfactant Mixtures

**Figure 4:**
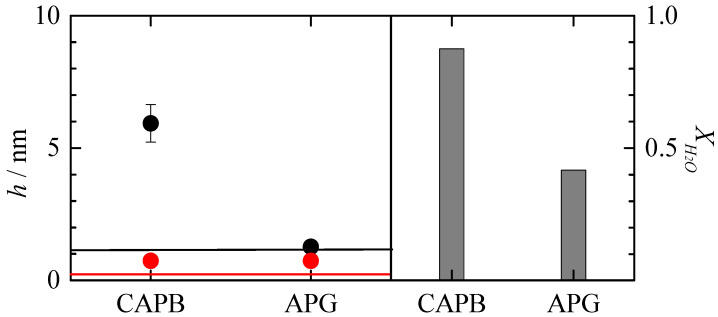
Acoustic (●) and optical thickness (●), and water content (bar charts) for the adsorption of binary PDADMAC–surfactant mixtures with the same concentration of the components as in Table 1. The solid lines represent the acoustic (―) and optical (―) thicknesses for the adsorption of polymer solutions with the same polymer concentration as that of the polymer–surfactant mixtures. Data of PDADMAC–CAPB mixtures were adapted from the work by Fernández-Peña et al. [18] under CC-BY license.

Before analyzing the effect of the dilution on the deposition of binary polyelectrolyte–surfactant mixtures, the adsorption of the concentrated one-phase mixtures was analyzed. Figure 4 shows a comparison of the optical, *h*_op_, and acoustic, *h*_ac_, thicknesses, as well as the degree of hydration of the layers obtained via adsorption from solutions of the concentrated binary PDADMAC–surfactant mixtures having a monophasic character in the composition given in Table 1, i.e., when the surfactant is CAPB or APG. Note that the data for PDADMAC–CAPB mixtures were extensively discussed in our previous publication [18].

The analysis of the behavior of the PDADMAC–CAPB mixtures shows that the electrostatic interaction between the carboxyl group of the surfactant and the quaternary ammonium groups of the polymer gives rise to more hydrated layers with higher thicknesses than those corresponding to the pure polymers (higher differences between the values of the acoustic and the optical thicknesses). In contrast, the neutral surfactant APG interacts with PDADMAC mainly via hydrophobic interactions. This polymer–surfactant interaction does not significantly modify the interaction of the polymers with the surface, and the formed complexes show adsorption similar to that of the bare PDADMAC. It should be noted that this is an unexpected finding as the mixture with APG is close to the onset of phase separation, and it would be expected that this mixture would show an enhanced deposition.

**Figure 5:**
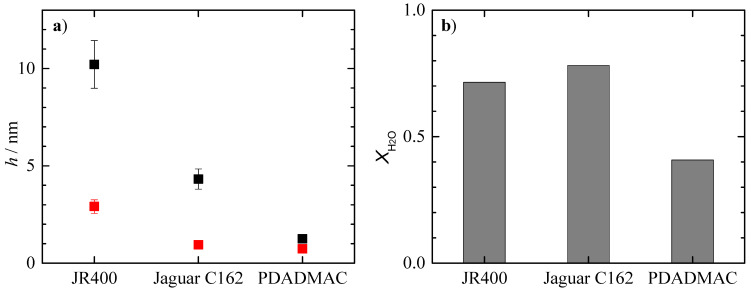
(**a**) Acoustic (■) and optical thickness (■) and (**b**) water content (bar charts) for the adsorption of binary PDADMAC–APG mixtures with the same concentration of the components as in Table 1.

The aim of this work is to analyze the potential use of cationic polysaccharides as substitutes for traditional conditioning polymers such as PDADMAC. Therefore, it is necessary to compare the adsorption of concentrated binary mixtures of PDADMAC with the surfactants and that corresponding to the mixtures containing the cationic polysaccharides. To this aim, Figure 5 shows a comparison of the optical and acoustic thicknesses of the adsorbed layers of the polymer-APG mixtures, as well as their degree of hydration. The results show that the mixtures of both polysaccharides with APG lead to the adsorption of layers with higher thicknesses than the PDADMAC–APG mixture, even though the hydration of the adsorbed layers is much higher than that corresponding to the layers of the mixture containing PDADMAC. This suggests that the nature of the polymer is the determining factor in the adsorption of this type of mixture. Thus, PDADMAC, which is a purely cationic polymer and has a relatively small size compared to the other polymers studied, adsorbs in a rod-like conformation, resulting in the formation of layers of relatively low thickness. In summary, the results suggest that PDADMAC can be replaced by both polysaccharides.

**Figure 6:**
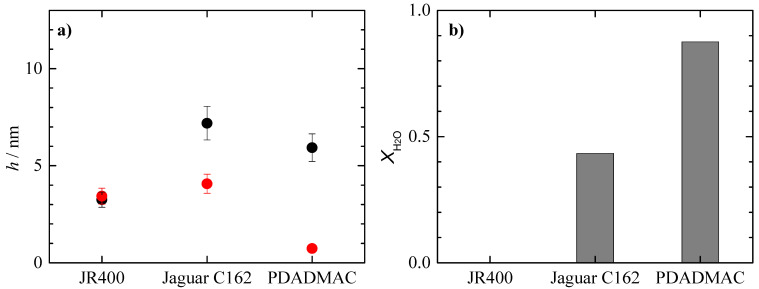
(**a**) Acoustic (●) and optical thickness (●) and (**b**) water content (bar charts) for the adsorption of binary PDADMAC–CAPB mixtures with the same concentration of the components as in Table 1. Data of PDADMAC–CAPB mixtures were adapted from the work by Fernández-Peña et al. [18] under CC-BY license.

For the mixtures containing CAPB, Figure 6 shows the thickness and degree of hydration of the adsorbed layers with respect to the different polymer–CAPB mixtures. The results suggest that PDADMAC can be replaced by Jaguar C162 but not by JR400. Although JR400 has a higher optical thickness than PDADMAC, the adsorbed layer is not hydrated, which is an essential requirement for conditioning purposes. Therefore, JR400 is not a good substitute for PDADMAC under the conditions of use. However, to confirm this point, the behavior of the concentrated binary polysaccharide–surfactant mixtures on dilution needs to be analyzed (results for mixtures containing PDADMAC were discussed in our previous publication [18]).

### 3.3. Study of the Dilution Enhanced Deposition of Concentrated Binary Polysaccharide–Surfactant Mixtures

**Figure 7:**
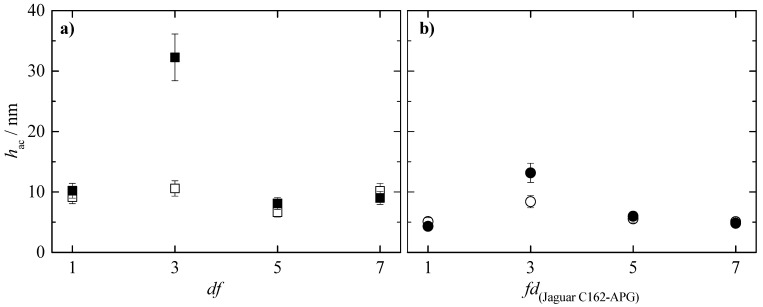
Acoustic thickness before (○ and □) and after (● and ■) rinsing/dilution for the adsorption of binary polysaccharide–APG mixtures obtained upon dilution at different values of *df* of binary polymer–surfactant mixtures with the same concentration of the components to that what is presented in the formulation of Table 1. (**a**) Mixtures with JR400. (**b**) Mixtures with Jaguar C162.

Considering our previous published work dealing with the behavior of concentrated mixtures containing PDADMAC [18], the study of the effect of the dilution on the deposition process of binary concentrated polymer–surfactant mixtures is focused on the mixtures of the two polysaccharides with CAPB and APG. Figure 7 shows the acoustic thicknesses corresponding to the adsorption process and subsequent rinsing of the polysaccharide–APG mixtures as a function of the *df* values (note that the rinsing process involved a dilution of the initial concentration by a factor close to 5).

**Figure 8:**
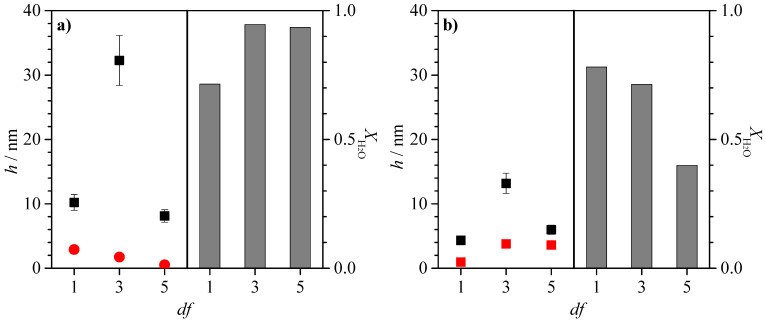
Comparison of the acoustic (■) and optical (● and ■) thickness for the deposition of polymer–APG layers and water content of the deposited layers (bar charts) for samples at different values of the dilution factor. (**a**) Mixtures with JR400. (**b**) Mixtures with Jaguar C162.

The results show that the acoustic thicknesses obtained via QCM-D are higher when JR400 is the polymer included in the polysaccharide–APG mixture p. Furthermore, it can be observed that in the mixtures of both polysaccharides with APG, when the concentrated mixture is diluted three times (*df* = 3), there is an increase in thickness after the rinsing process, with this increase being significantly higher in the JR400–APG mixture. This isolated increase in thickness in the system could initially be associated with the narrow phase separation region observed in Figure 2 and Figure 3, as the mixture is diluted in the washing process, forcing the system into the phase separation region. In turn, one would expect that the significant difference between the acoustic thicknesses after washing would be due to the higher hydrophobicity of JR400, which would facilitate the precipitation of the complexes and thus increase the thickness. However, Figure 8 shows that the optical thickness corresponding to the polysaccharide–APG mixture with *df* = 3 is higher in the binary system formed by Jaguar C162. This suggests that this difference in acoustic thickness is due to the hydration of the layer.

Given the non-ionic nature of the surfactant, the interaction of both polysaccharides with the APG must be via the formation of hydrogen bonds, and hydrophobic interactions may also occur between the tails of the surfactant and the hydrocarbon chain of the polymer. This means that the determining factor in the adsorption of the systems studied here is the type of monomers present in the polysaccharide chains, as this parameter is expected to condition the interaction between the complexes and the negatively charged surface. Therefore, in order to understand the behavior of the concentrated mixtures (*df* = 1), it must be taken into account that the structure of Jaguar C162 has twice as many quaternary ammonium-charged groups as JR400. Therefore, from the results shown in Figure 8 (*df* = 1), it is expected that the adsorption of the Jaguar C162–APG mixtures may occur with the chains in a more extended arrangement due to the double interaction of each polymer chain with the surface. Moreover, the adsorbed layer will consequently be more hydrated than the layer corresponding to the JR400–APG complexes because the high interchain repulsion prevents the densification process. On the other hand, the contribution of intra-chain repulsions would contribute to the adsorption in the above-mentioned conformations.

Having explained the adsorption phenomenology of the concentrated mixtures, if attention is focused on Figure 8, a decrease in the optical thickness with a dilution of the mixture can be observed, as well as a very high degree of hydration. Initially, such a sharp increase in the optical thickness at *df* = 3 could be attributed to the precipitation of the complexes after dilution, which occurs during the rinsing process as a consequence of the proximity of the system to the phase separation region. However, the decrease in the optical thickness with the dilution factor seems to indicate that the high value found for the acoustic thickness is only due to the high hydration of the adsorbed film. It is worth noting that the fact that there is such a marked increase in the acoustic thickness in both polysaccharide–APG systems could be due to a conformational change in the complexes as a consequence of dilution. However, given the proximity of the precipitation region, one would expect an increase in thickness as the mixture approaches the precipitation region, as in the case of the Jaguar C162 APG system (see Figure 8b). However, the results obtained for the JR400–APG system seem to indicate a different phenomenology, with the optical thickness decreasing as the *df* increases. The differences in behavior are difficult to explain on the basis of the similarities found in the dependence of turbidity on *df* (see Figure 3). A possible explanation could be related to differences in the precipitation kinetics of each system. For instance, in the Jaguar C162–APG system, precipitation appears to occur instantaneously during the rinsing, leading to an increase in thickness with df, whereas, in the JR400–APG system, precipitation appears to occur more slowly and is masked during the rinsing.

**Figure 9:**
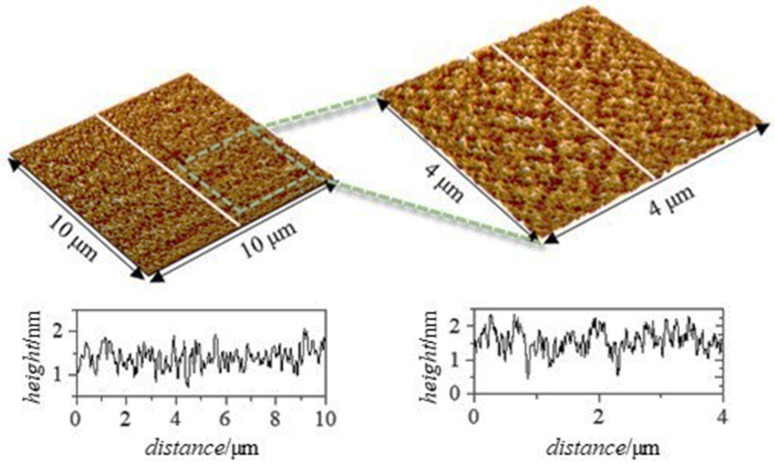
Experimental results obtained via AFM for a layer adsorbed from a JR400–APG solution of *df* = 3. Three-dimensional topographic images obtained over an area of 10 × 10 μm^2^ using a scanning resolution of 39 nm/pt (**left image**) and over an area of 4 × 4 μm^2^ using a scanning resolution of 15.6 nm/pt (**right image**). Below each image, the corresponding height profile performed along the white line is depicted.

In order to clarify the phenomenology involved in the adsorption of polysaccharide–APG mixtures with *df* = 3, an analysis of the topography of the layers formed on solid surfaces was carried out using atomic force microscopy (AFM). It is important to note that the results obtained in this study may differ from those obtained in solution since the AFM experiments were carried out under dry conditions, i.e., the samples were allowed to dry before measurements. To obtain the most accurate comparison, the height profiles obtained via AFM are compared with the thicknesses obtained via ellipsometry since the latter does not take into account the role of hydration water. Figure 9 shows two 3D topographic images obtained via AFM with their corresponding height profiles for a layer adsorbed on a solid surface from a solution of the JR400–APG mixture with *df* = 3.

The topographic images seem to indicate that the adsorption of the mixture leads to the formation of a homogeneous layer. They show small elevations, uniformly distributed over the whole sample, giving a 2D roughness of 0.14 nm according to ISO 4287:1997 (4 × 4 μm^2^ scan). On the other hand, the height profile of the adsorbed layer reaches a maximum value of ~2 nm, comparable to the thickness obtained via ellipsometry. This fact seems to indicate that there is no more adsorbed material under the topographic image obtained but that the zero obtained in the height profile is the solid silicon.

**Figure 10:**
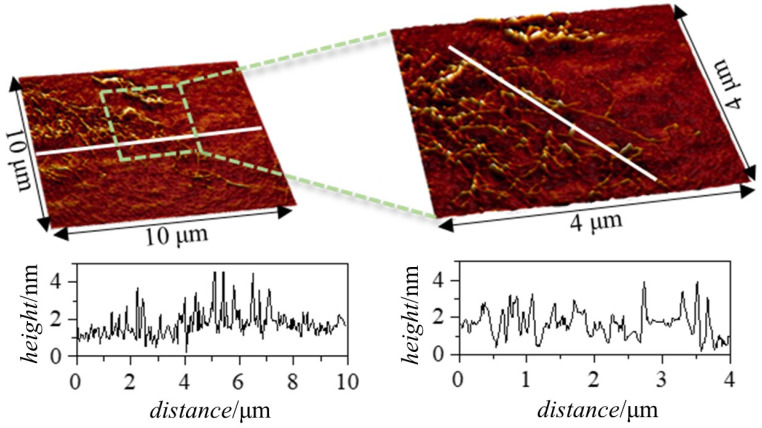
Experimental results obtained via AFM for a layer adsorbed from a Jaguar C162-APG solution of *df* = 3. Three-dimensional topographic images obtained over an area of 10 × 10 μm^2^ using a scanning resolution of 39 nm/pt (**left image**) and over an area of 4 × 4 μm^2^ using a scanning resolution of 15.6 nm/pt (**right image**). Below each image, the corresponding height profile performed along the white line is depicted.

The 3D images in Figure 10, corresponding to the adsorbed layers for a Jaguar C162–APG mixture with *df* = 3, show a much more heterogeneous appearance than the layers obtained for the mixture previously studied. In these images, filamentary structures can be observed, which could be associated with Jaguar C162–APG complexes deposited on a monolayer adsorbed directly on the surface.

In order to compare the roughness between the area where the filamentary structures appear and the area where they do not appear, 2D roughness parameters corresponding to an area of 16 µm^2^ were obtained. Firstly, the roughness of the area where the filamentary structures appear takes on a value of around 0.50 nm, while the area where no filamentary structures are present has a lower roughness (0.28 nm). These roughness results seem to indicate that the area without filamentary structures is also coated. This conclusion is drawn considering that the roughness of the coating is higher than that of silicon (0.1–0.17 nm in a 1 × 1 µm^2^ scan). In terms of the height profiles obtained, two types of profiles can be clearly distinguished. On the one hand, there are regions of the profile where the average height is 4–5 nm, in agreement with the results obtained via ellipsometry, and on the other hand, there are regions with a more homogeneous distribution of the adsorbed material, where the height profile oscillates around 1–2 nm.

**Figure 11:**
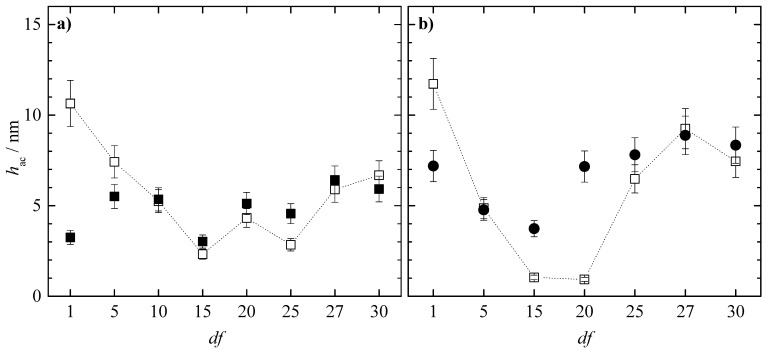
Acoustic thickness before (□) and after (● and ■) rinsing/dilution for the adsorption of binary polysaccharide–CAPB mixtures obtained upon dilution at different values of *df* of binary polymer–surfactant mixtures with the same concentration of the components to that what is presented in the formulation of Table 1. (**a**) Mixtures with JR400. (**b**) Mixtures with Jaguar C162.

The adsorption process of the polysaccharide–CAPB mixtures shows the expected trend based on studies of the dilution precipitation process. As observed in Figure 11, when the concentrated mixtures are diluted (*df* = 1), there is initially a decrease in adsorption, which would be consistent with the decrease in concentration in the solution. Furthermore, in the region corresponding to low *df* values, for the mixtures of CAPB with both polysaccharides, there is a decrease in the acoustic thickness with the washing process, which again can be associated with a reduction in the amount of adsorbed material. It should be noted that the dependencies found for the thickness of the Jaguar C162–CAPB and JR400–CAPB mixtures show some differences. Analyzing the case of the mixtures where the polymer is JR400, it is observed that from *df* = 15, the system starts to show an increase in adsorption with dilution. On the other hand, the amount of adsorbed material after washing also shows a slight increase with *df*. The combined analysis of these data seems to indicate that although the precipitation region has not been located for this system, the improvement in adsorption may be associated with the proximity of the precipitation region. This suggests that the precipitation kinetics for the system under consideration are very slow. Therefore, no macroscopic evidence was observed in the solutions. Similar behavior is found for the Jaguar C162–CAPB mixtures, although the appearance of evidence associated with the phenomenon of precipitation-enhanced adsorption is more clearly observed. For cosmetic applications, if the precipitation region is relatively far from the usual conditions of use of the commercial product, this would allow a reduction in the polymer content and surfactant concentration in the formulations, which could be advantageous both from an economic point of view. However, the role of slow precipitation kinetics could be detrimental as it would separate the time scales of the physical process of interest from the actual application process of the formulation. Therefore, a detailed evaluation of this process would be necessary in the future.

On the basis of the results shown in Figure 11, it appears that the Jaguar C162–CAPB mixtures show higher adsorptions than the analogous mixtures containing JR400. However, in order to draw conclusions from these results, it is necessary to carry out the adsorption study with ellipsometry to check whether this difference in adsorption is mainly related to water retention and not to the adsorption of a higher number of polysaccharide–surfactant complexes.

**Figure 12:**
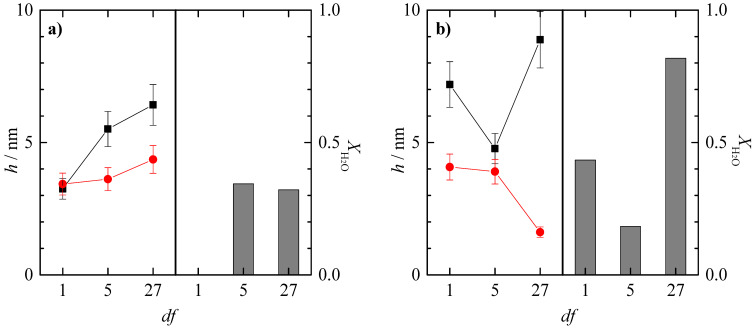
Comparison of the acoustic (■) and optical (●) thickness for the deposition of polymer–CAPB layers and water content of the deposited layers (bar charts) for samples at different values of the dilution factor. (**a**) Mixtures with JR400. (**b**) Mixtures with Jaguar C162.

Analyzing the adsorption of the concentrated polysaccharide–CAPB mixtures (*df* = 1) shown in Figure 12, it appears that the adsorption of the mixtures with Jaguar C162 is higher than the adsorption of the mixtures with JR400, and the opposite effect to that described for the polysaccharide–APG mixtures (*df* = 1) can be observed for mixtures containing CAPB. In this case, due to the zwitterionic nature of CAPB, there is an electrostatic interaction between the quaternary ammonium groups of the polysaccharides and the carboxyl groups of the surfactant. Since the structure of JR400 has one less quaternary ammonium group than Jaguar C162, the scenario observed in Figure 12a, where a non-hydrated adsorbed layer of the JR400–CAPB system is observed (*df* = 1), is expected. The reduction in electrostatic repulsion allows the quaternary ammonium groups of JR400, which are neither shielded by the salt nor interact with the surfactant, to interact with the solid surface, forming a very dense adsorbed layer without the possibility of incorporating water molecules. On the other hand, in the case of the Jaguar C162–CAPB mixture, due to its second charged group, there must be a high electrostatic repulsion between the different polymer chains, which allows the incorporation of water molecules into the adsorbed layer.

**Figure 13:**
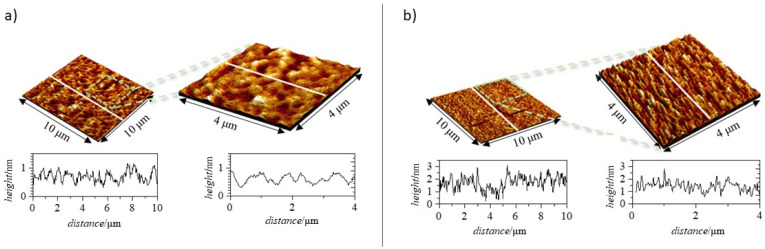
Experimental results obtained via AFM for layer adsorbed from polysaccharide–CAPB solutions of *df* = 27. Three-dimensional topographic images obtained over an area of 10 × 10 μm^2^ using a scanning resolution of 39 nm/pt (**left image**) and over an area of 4 × 4 μm^2^ using a scanning resolution of 15.6 nm/pt (**right image**). Below each image, the corresponding height profile performed along the white line is depicted. (**a**) JR400–APG mixtures. (**b**) Jaguar C162–APG mixtures.

In relation to the adsorption of the dilutions of the JR400–CAPB mixture shown in Figure 12a, an increase in adsorption with dilution is observed as a consequence of approaching the precipitation region. For the Jaguar C162–CAPB mixtures (Figure 12b), the densification of the adsorbed layer occurs at *df* = 5, while at *df* = 27, the system seems to leave the precipitation region as the adsorption decreases significantly, and the hydration of the layer becomes very high. The topographical analysis of the adsorbed layers for the polysaccharide–CAPB mixtures obtained via AFM is shown in Figure 13.

In both systems studied, the presence of ridges can be observed along the entire adsorbed layer. These grooves are elongated and narrower in the Jaguar C162–CAPB mixture with *df* = 27, which implies a modification of the roughness of the sample. In the Jaguar C162–CAPB layers, the roughness reaches a value of about 0.21 nm, while the mixtures with JR400 have a roughness of 0.11 nm (4 × 4 μm^2^ sweep). In turn, the height profiles of the JR400 mixture differ from the optical thicknesses, which is a sign that the zero of the height profile is still the material under investigation, i.e., the layer is so homogeneous and dense that only the surface of the sample can be observed. In contrast, the height profile of the Jaguar C162–CAPB layers coincides with the thickness obtained via ellipsometry, which is to be expected given the rough topography.

## 4. Conclusions

The potential substitution of conventional conditioning cationic polymers with naturally derived polymers in cosmetic cleansing formulations is a promising avenue for the cosmetics industry. By opting for naturally derived, environmentally sustainable alternatives such as polysaccharides, manufacturers can respond to the growing demand for environmentally friendly products. These naturally derived polymers can offer several benefits, including biodegradability and reduced environmental impact. They can also offer improved compatibility with different hair types and a gentler, more natural conditioning effect. However, thorough research and testing are required to ensure the efficacy and stability of these substitutes, as well as their ability to deliver consistent results across a wide range of hair types and conditions. In this context, this work has been focused on investigating the physicochemical underpinnings of the performance of cosmetic shampoo formulations containing cationic polysaccharides as model hair conditioners. This has required the study of the dilution-induced deposition of concentrated mixtures of binary mixtures formed by cationic polysaccharides and two different surfactants, the neutral APG and the zwitterionic CAPB, which are commonly used in many shampoo and gel formulations. The study of these systems has shown that although the phase behavior on the dilution of the mixtures can be similar when they contain the same polysaccharide, strong differences can appear in relation to their deposition mediated via the dilution process. Therefore, the evaluation of the effectiveness of the formulations must take into account both the surfactant nature and the specific structure of the polymer considered.

It should be stressed that the results obtained show that polysaccharides are a promising alternative to conventional cationic polymers, such as PDADMAC. Dedicated protocols in vitro or in vivo on hair are ultimately needed to fully optimize complex formulations.

## Figures and Tables

**Table 1:** Concentration of the different components forming the concentrated binary polyelectrolyte–surfactant mixtures.

Component	Concentration	Type of Molecule
PDADMAC, JR400 or Jaguar C162	0.5% *w*/*w* ^1^	Polymers
SLES	186 mM	Surfactants
CAPB	204 mM	
AKYPO	136 mM	
APG	64 mM	

^1^ The concentration of PDADMAC in terms of monomer units is 30 mM, while in the case of JR400 and Jaguar C162, it is 8 mM.

**Table 2:** Single-phase or phase-separated character of the different concentrated binary polyelectrolyte–surfactant mixtures studied in this work.

Polyelectrolyte	Surfactant	Type of Sample
PDADMAC	SLES	2ϕ
	AKYPO	2ϕ
	CAPB	1ϕ
	APG	1ϕ
JR400	SLES	2ϕ
	AKYPO	2ϕ
	CAPB	1ϕ
	APG	1ϕ
Jaguar C162	SLES	2ϕ
	AKYPO	2ϕ
	CAPB	1ϕ
	APG	1ϕ

## Data Availability

Data is available upon request.
